# Morphological and Molecular Characterization of *Oscheius saproxylicus* sp. n. (Rhabditida, Rhabditidae) From Decaying Wood in Spain, With New Insights into the Phylogeny of the Genus and a Revision of its Taxonomy

**DOI:** 10.21307/jofnem-2019-053

**Published:** 2019-09-17

**Authors:** Joaquín Abolafia, Reyes Peña-Santiago

**Affiliations:** 1 Departamento de Biología Animal, Biología Vegetal y Ecología, Universidad de Jaén. Campus “Las Lagunillas” s/n. 23071-Jaén, Spain

**Keywords:** 18S rDNA, 28S rDNA, description, Iberian Peninsula, molecular analysis, morphology, new species, rhabditids, SEM, taxonomy

## Abstract

A new species of the genus *Oscheius*, *O. saproxylicus* sp. n., collected in decaying wood obtained from an orchard in Southern Iberian Peninsula, is reported. A detailed description, including morphometrics, LM and SEM images, and molecular (18S and 28S rDNA genes) information is provided. The female is characterized by a moderately long body, lateral field with three longitudinal ridges, midbody vulva, and conical tail with acute tip. It was distinguished from its closest relative, *O. dolichura*, by a shorter tail and longer rectum. The male was not found. Morphological and molecular data support its belonging to *Dolichura*-group. Molecular analyses show that both *Insectivorus* and *Dolichura* groups are related to each other, being proposed as subgenera of the genus *Oscheius* as *Oscheius* and *Dolichorhabditis.* Finally, an updated taxonomy of the genus is presented, with generic and subgeneric diagnoses, list of species and a key to their identification.

Andrássy (1976) proposed the new genus *Oscheius* under Rhabditidae (Örley, 1880), Rhabditinae (Örley, 1880), with *O. insectivorus* (=*Rhabditis insectivora* Körner *in* Osche, 1952) as its type and only species. *Oscheius* was distinguished from other Rhabtidinae by its unusually short buccal tube, about as long as wide, and the absence of median pharyngeal swelling. Later, the same author (1983, 1984) transferred a second species, *O. koerneri* (=*Rhabditis koerneri* Osche, 1952), to the genus and provided its diagnosis.

Sudhaus and Hooper (1994) provided new ideas about the taxonomy and the phylogeny of several rhabditid species: (i) accepted *Oscheius* as a subgenus of *Rhabditis*, (ii) regarded it as a monophyletic taxon based on three synapomorphies (long female rectum, terminal duct of the excretory system forwards coiled and with heavily sclerotized wall, and several features of spicule shape), (iii) considered *Dolichorhabditis* (Andrássy, 1983) as a junior synonym of *Oscheius*, and (iv) distinguished two species groups within the subgenus. One of these groups, the *Insectivorus*-group, included seven species with leptoderan or pseudopeloderan bursa (male tail with a filiform part standing out behind the bursa, a plesiomorphic state), and spicules with crochet needle shaped tip (apomorphic state). The second group, the *Dolichura*-group, with five species previously classified under *Dolichorhabditis* and having peloderan bursa (lacking the filiform part, an apomorphic condition) and spicules with thin tubular tip (plesiomorphic condition). Andrássy (2005) reinstated the generic range for *Oscheius*, listed a total of eight species under it, and distinguished it from *Dolichorhabditis*, with ten valid species, by several differences in stomatal teeth, bursa and spicules. The separation of both genera has been accepted in several contributions (Abolafia and Peña-Santiago, 2010; Gorgadze, 2010), but Sudhaus (2011) and Tabassum et al. (2016) maintained *Dolichorhabditis* as junior synonym of *Oscheius* as well the two monophyletic species groups within the latter.

Molecular data of *Oscheius sensu lato* species, many of them described during the last years, have been matter of analyses by several authors (Félix et al., 2001; Darby et al., 2011; Darsouei et al., 2014; Campos-Herrera et al., 2015; Torrini et al., 2015; Tabassum et al., 2016; Lima de Brida et al., 2017; Valizadeh et al., 2017; Zhou et al., 2017), resulting in the confirmation of the monophyly of the *Insectivorus*- and the *Dolichura*-group. Nonetheless, their nature as sister groups was not always corroborated (van Megen et al., 2009; Darsouei et al., 2014; Zhou et al., 2017).

An *Oscheius* population was collected in the course of a nematological survey conducted in southern Iberian soils. Its study revealed it belonged to a non-described form. The aims of this contribution are to characterize this material, to provide new insights on the phylogeny of the group, and to update its taxonomy.

## Materials and methods

### Nematode extraction and processing

Nematodes were collected from dead wood using a modified trays technique (Whitehead and Hemming, 1965), killed by heat, fixed in 4% formalin, transferred to pure glycerine following the Siddiqi’s (1964) method, and mounted on permanent glass slides. Moist, dead wood was maintained as a culture to extract specimens every several months.

### Light microscopy (LM)

Observations were made using a Leitz Laborlux S (Leitz, Wetzlar, Germany) and Nikon Eclipse 80i (Nikon, Tokio, Japan) microscopes. Measurements were taken with the Leitz microscope, which has a drawing tube (*camera lucida*) attached to it, and Demanian indices and other ratios calculated. Drawings were made using the Leitz microscope. Images were taken with the Nikon microscope that was provided with differential interference contrast (DIC) optics and Nikon Digital Sight DS-U1 camera. Micrographs were edited using Adobe^®^ Photoshop^®^ CS. The terminology used for the morphology of stoma and spicules follows the proposals by De Ley et al. (1995) and Abolafia and Peña-Santiago (2017), respectively.

### Scanning Electron Microscopy (SEM)

Specimens preserved in glycerine were selected for observation under SEM according to Abolafia (2015). They were hydrated in distilled water, dehydrated in a graded ethanol-acetone series, critical point dried, coated with gold, and observed with a Zeiss Merlin microscope (5 kV) (Zeiss, Oberkochen, Germany).

### DNA Extraction, PCR and Sequencing

Nematode DNA was extracted from single fresh individuals using the proteinase K protocol and PCR assays as described Castillo et al. (2003) somewhat modified. Specimen was cut in small pieces using a sterilized dental needle on a clean slide with 18 ml of AE buffer (10 mM Tris-Cl + 0.5 mM EDTA; pH 9.0), transferred to a microtube and adding 2 μl proteinase K (700 μg/ml) (Roche, Basel, Switzerland), and stored to –80°C within 15 min (for several days). The microtubes were incubated at 65°C (1 hr), then at 95°C (15 min). The microtube was centrifuged to 13,000 r.p.m. (or 15,900 × *g*) for 3 min. and 2 μl of the supernatant extracted DNA was transferred to a microtube containing: 2.5 μl ×10 PCR reaction buffer, 5 μl Q-solution ×5, 0.5 μl dNTPs mixture (10 mM each), 1 μl of each primer (10 mM), 0.2 μl Taq DNA Polymerase (Qiagen, Venlo, The Netherlands) and ddH2O to a final volume of 25 μl. The primers used for amplification of the D2-D3 region of 28S rRNA gene were the D2A (5′-ACAAGTACCGTGAGGGAAAGTTG-3′) and the D3B (5′-TCGGAAGGAACCAGCTACTA-3′) primers (De Ley et al., 1999). PCR cycle conditions were as follows: one cycle of 94°C for 3 min., followed by 35 cycles of 94°C for 1 min. + annealing temperature of 55°C for 45 s + 72°C for 2 min., and finally one cycle of 72°C for 10 min. After DNA amplification, 5 μl of product was loaded on a 1% agarose gel in 0.5% Tris-acetate-EDTA (40 mM Tris, 20 mM glacial acetic acid and 2 mM EDTA; pH = 8) to verify the amplification using a electrophoresis system (Labnet Gel XL Ultra V–2, Progen Scientific, London, UK). The bands were stained with RedSafe (×20,000) previously added to the agarose gel solution. PCR products were purified using the QIAquick PCR purification kit (Qiagen, Venlo, The Netherlands), quantified using a spectrophotometer (Synergy HT, BioTek, Winooski, USA) and used for direct sequencing in both directions using the primers referred to above. The sequencing reactions were performed at “Centro de Instrumentación Científico-Técnica (CICT)” of the University of Jaén (Spain) using an Applied Biosystems Hitachi 3500 Genetic Analyzer. The sequences obtained were submitted to the GenBank database.

### Phylogenetic analyses

For phylogenetic relationships, analyses were based on 18S and 28S rDNA. The newly obtained sequences were manually edited using BioEdit 7.2.6 (Hall, 1999) and aligned with another 18S or 28S rRNA gene sequences available in GenBank using Muscle alignment tool implemented in the MEGA7 (Kumar et al., 2016). The ambiguously aligned parts and divergent regions were known using the online version of Gblocks 0.91b (Castresana, 2000) (http://molevol.cmima.csic.es/castresana/Gblocks_server.html) and were removed from the alignments using MEGA7. The best-fit model of nucleotide substitution used for the phylogenetic analysis was statistically selected using jModelTest 2.1.10 (Darriba et al., 2012). Phylogenetic tree was generated with Bayesian inference method using MrBayes 3.2.6 (Huelsenbeck and Ronquist, 2001; Ronquist and Huelsenbeck, 2003). *Myolaimus byersi* (KU180665 for 18S and KU180676 for 28S) was chosen as outgroup according to previous results by Kanzaki et al. (2009). The analysis under GTR+I+G model was initiated with a random starting tree and run with the Markov Chain Monte Carlo (MCMC) for 1 × 10^6^ generations. The tree was visualized and saved with FigTree 1.4.3 (Rambaut, 2014).

### Descriptions

*Oscheius saproxylicus* sp. n.^1^

(Figs. 1–3).

**Figure 1:**
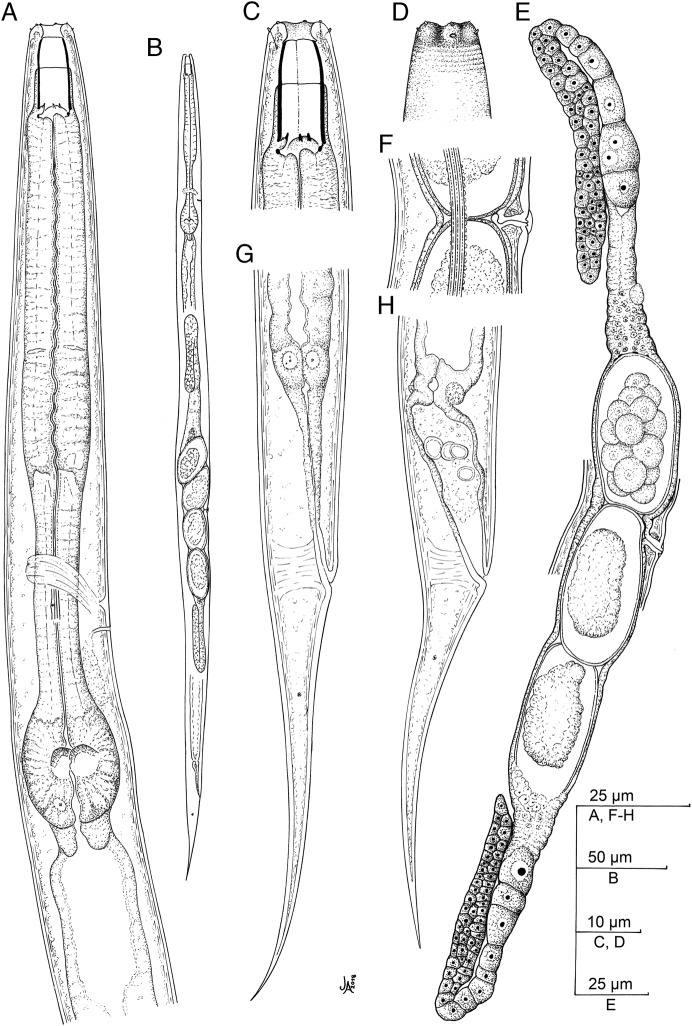
*Oscheius saproxylicus* sp. n. (line drawing). (A) Neck; (B): Entire female; (C) Stoma; (D) Lip region; (E) Genital system; (F) Vagina; (G, H) Female tail.

**Figure 2:**
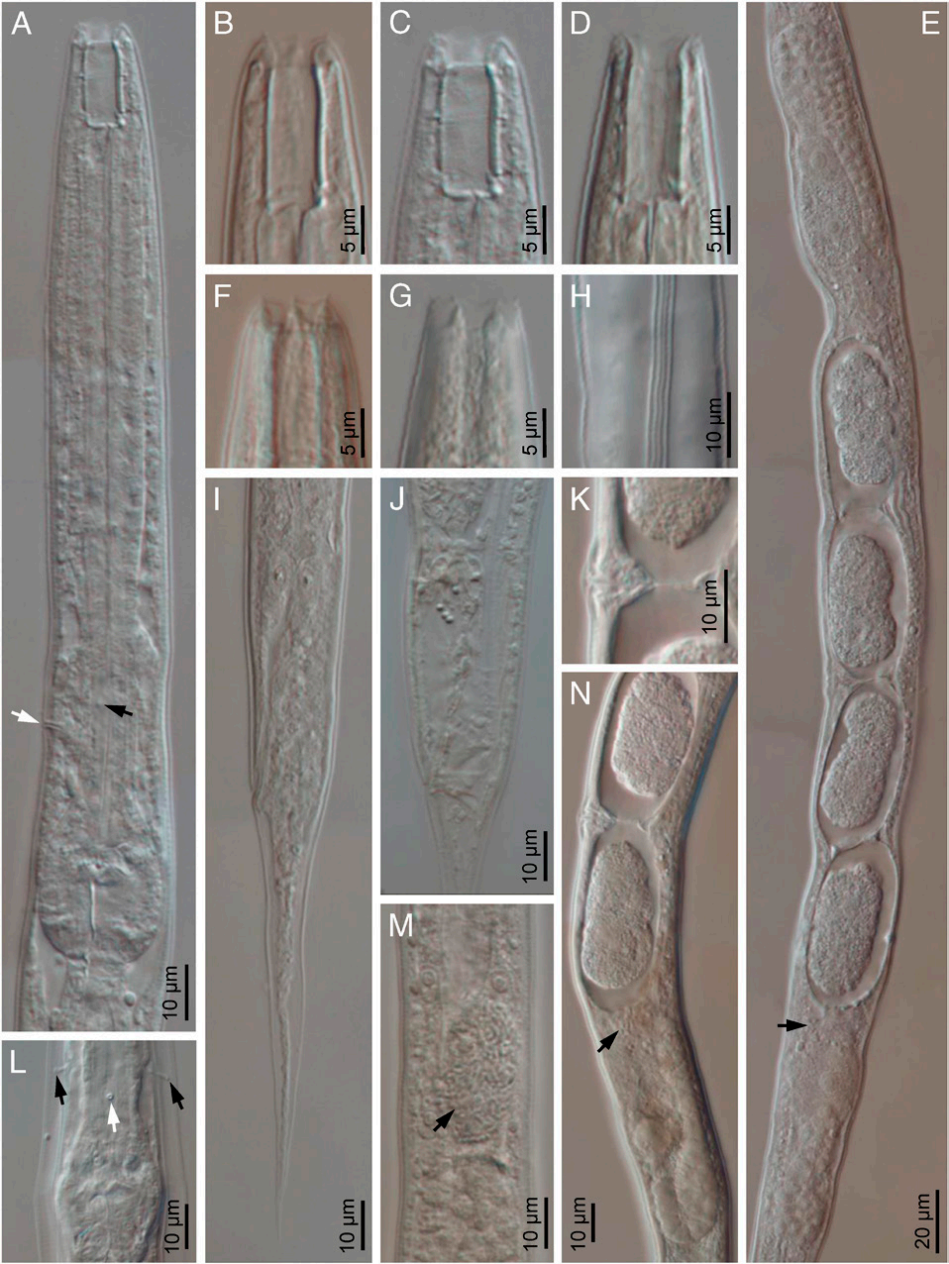
*Oscheius saproxylicus* sp. n. (light microscopy, female). (A) Neck; (B-D) Stoma in lateral (B, C) and dorso-ventral (D) views; (E, N) Reproductive system (arrow at spermatozoa); (F, G) Lip region in lateral and ventral views, respectively; (H) Lateral field; (I, J) Posterior end with rectum empty and swollen, respectively; (K) Vagina; (L) Excretory pore in ventral view (white arrow) and deirids (black arrow); (M) Intestine cell with microsporidia (arrow).

**Figure 3:**
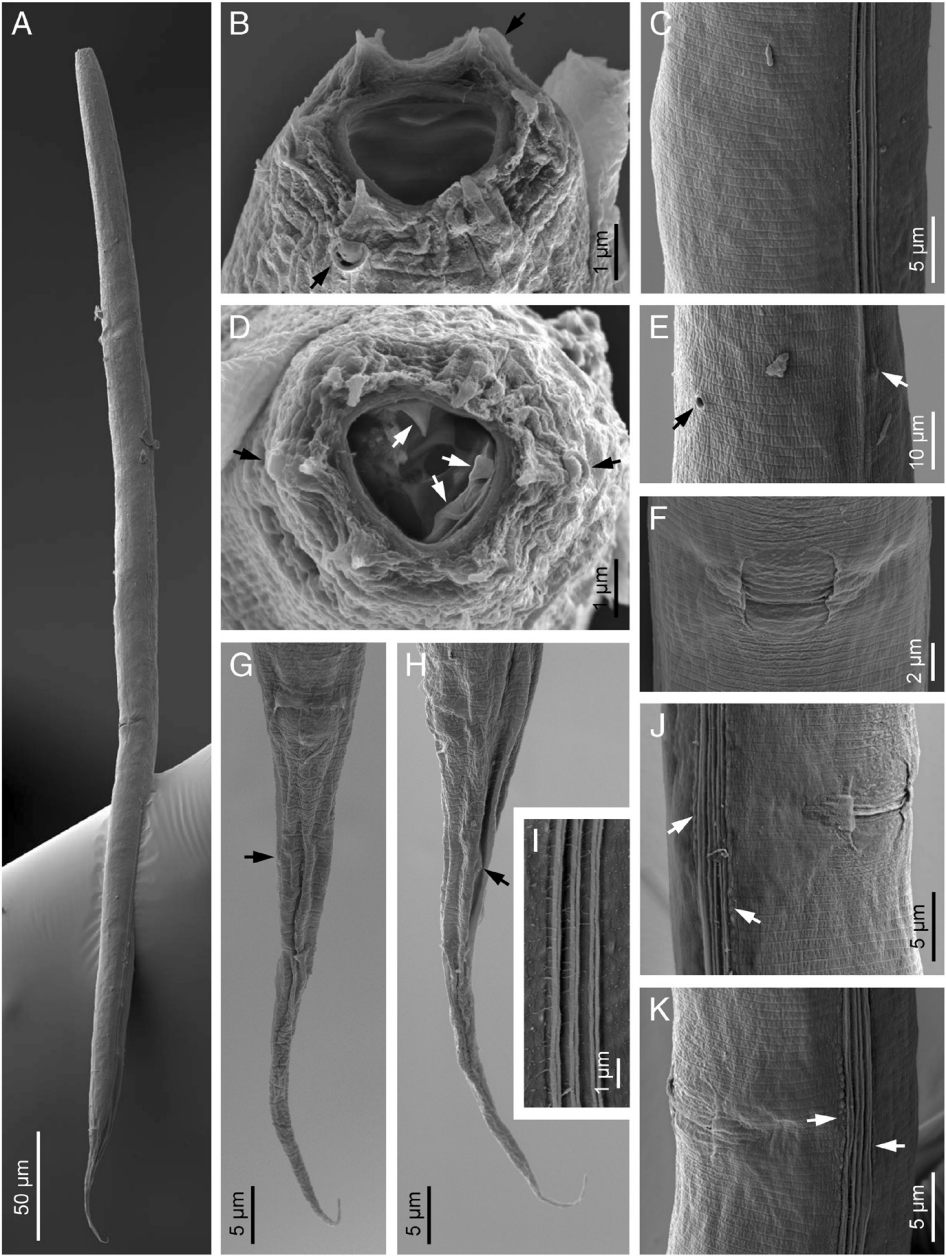
*Oscheius saproxylicus* sp. n. (scanning electron microscopy, female). (A) Entire body; (B, D) Lip region in subfrontal and frontal views, respectively (black arrows at amphid and white arrows pointing the metastegostomatal teeth, up: dorsal tooth, down: sublateral teeth); (C) Cuticle at midbody; E: Excretory pore (black arrow) and deirid (white arrow); (F, J, K) Vulval region at ventral, sublateral right and left views, respectively (arrows at lineal warts of the lateral fields); (G, H) Tail in ventral and lateral views, respectively (arrow at phasmid); (I) Lateral field.

#### Material examined

Fifty one females in generally acceptable state of preservation.

#### Measurements

See Table 1.

**Table 1.** Morphometrics of *Oscheius saproxylicus* sp. n. Measurements in μm and in the form: mean ± standard deviation (range) where appropriate.

Locality	Puente de la Sierra	
Province	Jaén	
Habitat	Dead wood	
n	Holotype female	Paratypes 50 females
Body length	829	837 ± 78.4 (669–994)
a	37.7	29.6 ± 3.2 (23.9–37.1)
b	5.1	5.0 ± 0.5 (3.9–6.5)
c	11.5	11.0 ± 1.2 (8.7–13.8)
c'	5.5	5.5 ± 0.5 (5.0–7.0)
V	57	54.8 ± 2.4 (48–59)
Lip region width	9	9.6 ± 0.5 (9–10)
Stoma length	18	18.7 ± 1.0 (17–21)
Stoma width	5	5.8 ± 0.6 (5–7)
Pharyngeal corpus length	72	75.9 ± 6.5 (68–96)
Isthmus length	48	47.7 ± 3.9 (39–54)
Bulbus length	26	26.6 ± 1.4 (24–29)
Pharynx length	146	149 ± 9.4 (113–165)
Neck length	164	168 ± 9.6 (131–182)
Body diameter at neck base	22	23.9 ± 1.7 (20–28)
Body diameter at midbody	22	28.5 ± 3.4 (21–37)
Vulva - anterior end	469	459 ± 49.0 (366–549)
Rectum length	48	44.8 ± 3.8 (40–54)
Anal body diameter	13	13.9 ± 1.1 (11–16)
Tail length	72	76.3 ± 5.0 (67–88)
Phasmid - anus distance	19	23.9 ± 3.4 (20–30)

Notes: Demanian indices (de Man, 1880): a = body length/body diameter; b = body length/pharynx length; c = body length/tail length; c’ = tail length/anal body diameter; V = (distance from anterior region to vulva/body length) × 100.

Description.

#### Female

Moderately slender to slender (*a* = 24–37) nematodes of small size, body 0.67 to 0.99 mm long. Upon fixation, habitus straight or somewhat curved ventrad. Cuticle 1 µm thick, nearly smooth under LM, but bearing very fine transverse striation when observed with SEM. Lateral field with three longitudinal ridges (alae), 3 to 6 µm broad or occupying one-tenth to one-fifth (11–19%) of mid-body diameter, and extending to phasmids. Lip region continuous with the adjacent body: lips rounded, separated by deep, U-shaped axils with six rounded labial and four acute cephalic sensilla. Amphids conspicuous, oval, with swollen margin. Stoma typical rhabditoid, 1.8 to 2.1 times the lip region width long or 2.6 to 4.0 times longer than broad: cheilostom lacking refringent rhabdia; gymno-promesostegostom (buccal tube) barrel-shaped, with gymnostom slightly narrower at its anterior part, glottoid apparatus of metastegostom with minute denticles, two per valve, and telostegostom consisting of small rounded rhabdia. Pharynx rhabditoid: subcylindrical corpus 1.3 to 2.2 times longer than isthmus, and with not swollen metacorpus; isthmus robust, visibly thinner than metacorpus; basal bulb ovoid, with well-developed valvular apparatus. Cardia conoid, surrounded by intestinal tissue. Nerve ring located at 103 to 132 µm or 65 to 75% of neck length from the anterior end, at level of about middle isthmus. Excretory pore at 97 to 152 µm or 63 to 89% of neck length from the anterior end, at level of the middle or posterior part of isthmus. Deirids hardly in front of excretory pore, at 112 to 146 µm or 63 to 86% of neck length, at level of about middle isthmus. Intestine lacking any distinct differentiation, but its wall becoming thinner at cardiac part, and its cells often associated/infected with microsporidia spores (cf. Nishikori et al., 2018). Three large gland-like cells are present around the intestine-rectum junction. Rectum very long, 3.0 to 4.2 times the anal body diam. Reproductive system didelphic-amphidelphic, the anterior branch in dextral position to intestine and the posterior one in sinistral position: ovaries 64 to 156 µm long, with a flexure at their middle; oviducts short, 32 to 68 µm long, barely discernible, distally differentiated in a more or less spherical spermatheca with small female sperm cells inside; uteri very variable in length, 32 to 146 µm long or 1.5 to 4.9 times the corresponding body diameter, tubular, often containing several eggs in different developmental stages; vagina 6 to 9 µm long, extending inwards to 22 to 33% of body diameter; vulva not protruding, displaying lateral epiptygma. Tail conical-elongate with fine acute terminus, 1.4 to 2.2 times the rectum long. Phasmids located at 20 to 30 µm or 25 to 39% of tail length from anus.

#### Male

Unknown.

### Remarks

During the culture of nematodes in dead wood under wet conditions, hundreds of *Oscheius saproxylicus* sp. n. specimens were obtained from a moist, dead wood culture. One hundred females and numerous juveniles were observed, but males were not found. Females exhibited consistent morphology. Generation of males by starvation in culture plate (Carta and Osbrink, 2005) could not be done. The presence of very small cells at uteri, which could represent hermaphrodite sperm (LaMunyon and Ward, 1998; Woodruff et al., 2010; Ellis and Schärer, 2014; Ellis and Wei, 2015), and the absence of males could indicate the evidence of hermaphroditism in this species.

### Molecular characterization

One 923 bp 18S rDNA sequence (GenBank accession number MK959600) and three identical 637 bp without changes or deletions each other 28S rDNA sequences (GenBank accession numbers MK959601-MK959603) were obtained from three specimens. Both trees show *O. dolichura* as the sister species of *O. saproxylicus* sp. n. With respect to *O. dolichura*, the 18S fragment (with 914 bp), show four changes or deletions while the 28S fragments (with 637 bp) show one change.

### Diagnosis

The new species is characterized by its 0.67 to 0.99 mm long body, cuticle with very fine transverse striation, lateral field with three longitudinal alae, lip region 9 to 10 µm broad and consisting of six separated lips, stoma 17–21 × 5–7 µm with barrel-shaped gymnoprostegostom, neck 131 to 182 µm long, pharynx cylindrical with metacorpus not swollen and broad isthmus, excretory pore and deirids at isthmus level, female reproductive system didelphic-amphidelphic, *V* = 48 to 59, rectum 3.0 to 4.2 anal body widths long, female tail conical with acute tip (67–88 µm, *c* = 8.7–13.8, *c*’ = 5.0–7.0), and male unknown.

### Relationships

**Figure 4:**
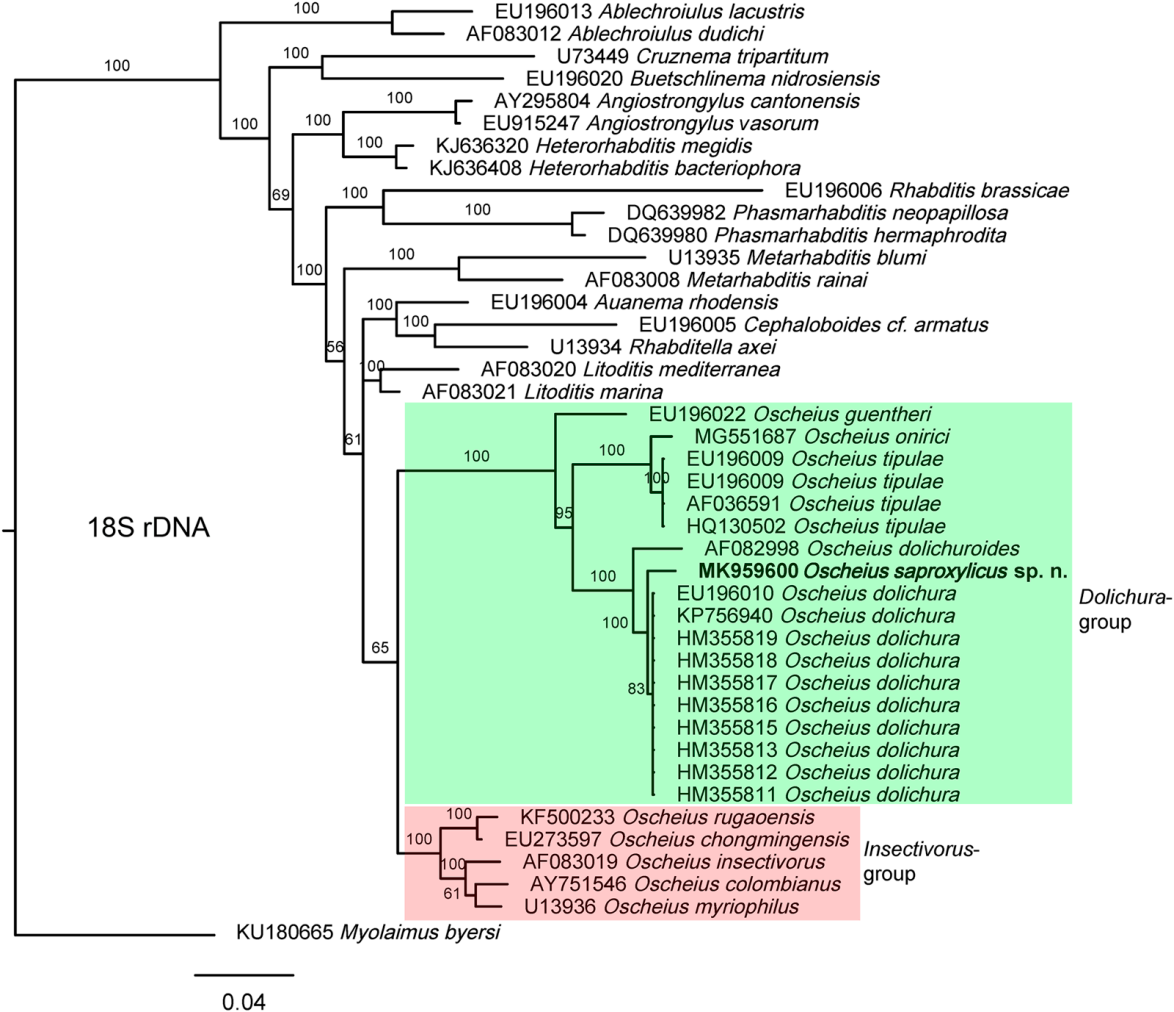
Bayesian Inference tree from known and the newly sequenced *Oscheius saproxylicus* sp. n. based on sequences of the 18S rDNA region. Bayesian posterior probabilities (%) are given for each clade. Scale bar shows the number of substitutions per site.

**Figure 5:**
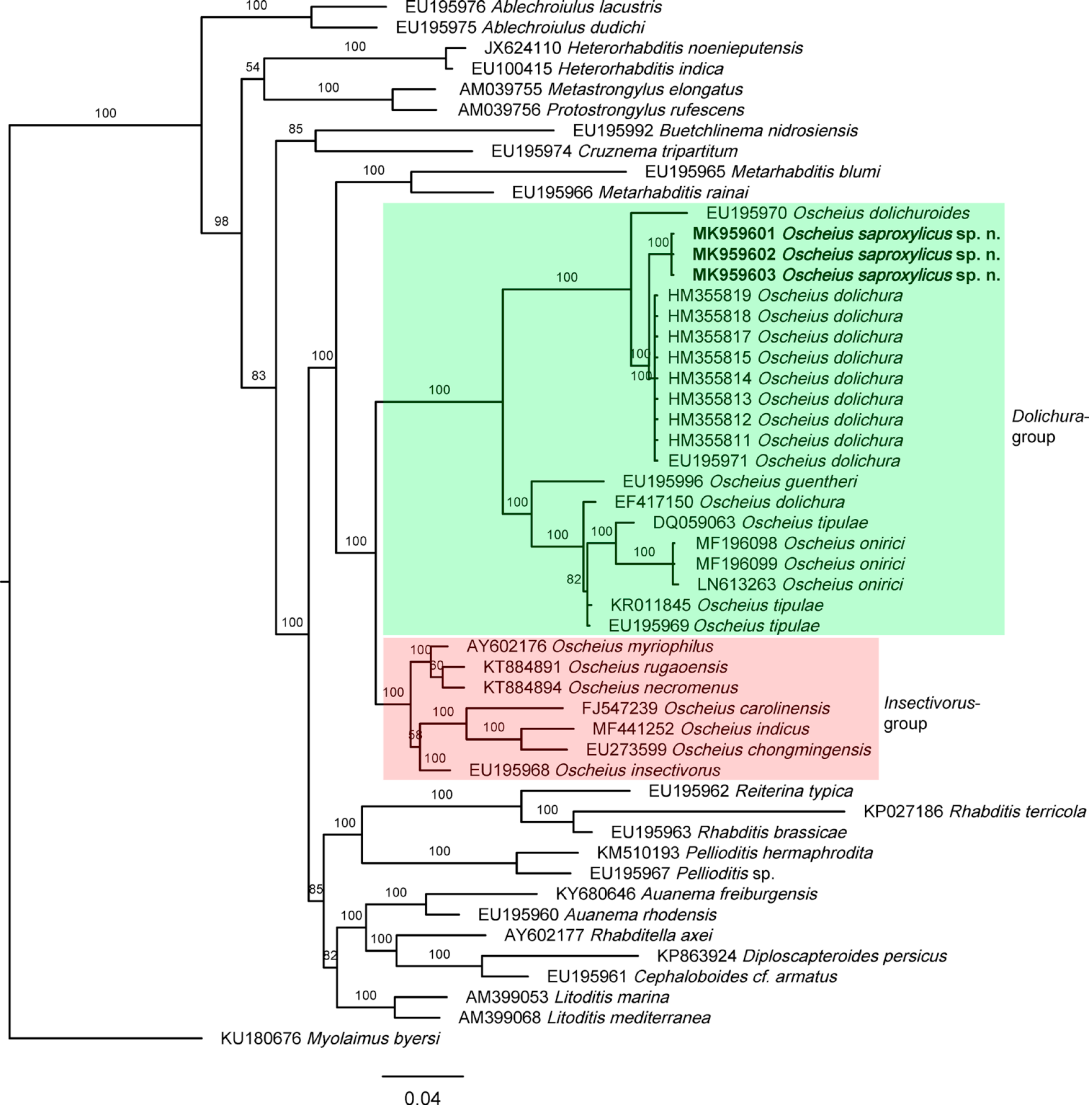
Bayesian Inference tree from known and the newly sequenced *Oscheius saproxylicus* sp. n. based on sequences of the 28S rDNA region. Bayesian posterior probabilities (%) are given for each clade. Scale bar shows the number of substitutions per site.

The absence of males in this population of the new species does not allow its classification under the *Insectivorus*- or the *Dolichura-*group with total certainty, but its general morphology better fits the representatives of *Dolichura*-group. Evolutionary relationships derived phylogenetic trees (Figs. 4, 5) show that *O. saproxylicus* sp. n. forms part of a highly supported clade also including *O. dolichura* and *O. dolichuroides*, two representatives of the *Dolichura*-group. Within this group, the new species is similar to *O. pseudodolichura* (Körner in Osche, 1952) (Sudhaus and Hooper, 1994) from Germany and *O. tereticorpus* (Kito and Ohyama, 2008) from Antarctica, especially in its slender gymnostom, long rectum and tail shape. Nonetheless, it differs from *O. pseudodolichura* by its smaller general size (body 0.67–0.99 vs 0.92–1.22 mm long), lip region nearly continuous (vs offset), not swollen (vs swollen) metacorpus, and excretory pore location (isthmus vs basal bulb level). From *O. tereticorpus* by the presence (vs absence) of cuticle warts in parallel to lateral field at vulva level, slightly broader stoma (5–7 vs 4–5 µm) with gymnostom somewhat shorter than the promesostegostom, metacorpus lacking inner valve-like structures, spermatheca simple vs developing a dorsal sac, and protruding anal lips.

It also resembles *O. debilicauda* (Fuchs, 1937) n. comb. from Germany, *O. dolichura* (Schneider, 1866) (Sudhaus and Hooper, 1994), *O. dolichuroides* (Anderson and Sudhaus, 1985) Sudhaus and Hooper (1994) from Kenya, and *O. janeti* (De Lacaze-Duthiers in Janet, 1893) (Sudhaus, 2011) from France, but it can be distinguished from these by the gymnostom tapering at its anterior part (vs more or less uniformly broad throughout its length). Besides, it is separated from *O. debilicauda* by the relative size of gymnostom (as long as vs one-third of the promesostegostom), and longer female rectum (3.0–4.2 vs 1.4 ABW). From *O. dolichura*, a widespread species (Schneider, 1866; Bütschli, 1873; Örley, 1886; Maupas, 1900; Micoletzky, 1922; Rahm, 1924; Völk, 1950; Andrássy, 1952, 1958, 2005; Wahab, 1962; Ali et al., 1973) by having comparatively shorter female tail (*c*’ = 5.0–7.0 vs *c*’ = 2.5–4.2), and longer female rectum (3.0–4.4 vs 2.2–3.0 ABW). From *O. dolichuroides* by its smaller general size (body 0.67–0.99 vs 1.17–1.58 mm long), comparatively shorter stoma (1.8–2.1 vs 4.4 times the lip region diam.), and longer female rectum (3.0–4.4 vs 2.2–3.3 ABW). From *O. janeti* by its longer female rectum (3.0–4.4 vs 1.9–2.2 ABW), comparatively longer female tail (*c* = 8.7–13.8 vs *c* = 15–17, *c*’ = 5.0–7.0 vs *c*’ = 2.6–3.5), and juvenile tail lacking a mucro (vs bearing a rattlesnake-like, annulated, acute mucro).

### Type locality and habitat

Spain, Jaén province, Jaén town, Puente de la Sierra (GPS coordinates: 37°42'36.5″N and 3°45'33.2″W, elevation 439 m), in association with decaying wood from dead white poplar trees present at the boundaries of an orchard.

### Type material

Fourty seven females (holotype and paratypes) deposited in Departamento de Biología Animal, Biología Vegetal y Ecología, Universidad de Jaén, Spain; four female paratypes deposited in nematode collection of the Swedish Museum of Natural History, Stockholm (Sweden).

## Phylogeny and Systematics of *Oscheius Sensu Lato*

Evolutionary relationships of *Oscheius* species have been previously analyzed with either traditional (morphological) or a modern (molecular) perspective, but an integrative approach is lacking yet. As mentioned in the introductory section, Andrássy (1976, 1984, 2005), on the basis of morphological data, defended the separation of *Osheius* from other Rhabditinae genera, especially from *Dolichorhabditis*, whereas Sudhaus and Hooper (1994; see more recently Sudhaus, 2011), by means of morphological cladistic analyses, advocated the synonymy of *Oscheius* and *Dolichorhabditis*. However he recognized two monophyletic species groups, the *Insectivorus*-group, including *Oscheius* species *sensu* Andrássy (op. cit.), and the *Dolichura*-group, including *Dolichorhabditis* species *sensu* Andrássy (op. cit.). Available molecular analyses (Ye et al., 2010, 2018; Darby et al., 2011; Liu et al., 2012; Zhang et al., 2012; Campos-Herrera et al., 2015; Torrini et al., 2015; Tabassum et al., 2016; Lima de Brida et al., 2017; Valizadeh et al., 2017) have repeatedly confirmed the monophyly of both *Insectivorus-* and *Dolichura*-groups, and most of them agree that these groups are sister groups. Nevertheless, two contributions (Darsouei et al., 2014; Zhou et al., 2017) do not support this idea.

The molecular analysis of 18S and 28S rDNA sequences of the new species herein described, whose results are presented in the trees of Figures 4 and 5, respectively, confirm the monophyly of both species groups as well as that they are sister groups. Thus, *O. saproxylicus* sp. n. sequences form a highly supported (100%) clade with several representatives of the *Dolichura-*group. The well-supported (100%) *Insectivorus* sister group joins it in a larger, highly supported (100%) clade in the 28S tree but moderately supported (65%) clade in the 18S tree. In its turn, the *Dolichura*-group/clade consists of three highly supported sub-clades that should be a matter of further analysis when more sequences become available. Internal relationships within the *Insectivorus*-group/clade cannot be elucidated yet.

Regarding the outer relationships of *Oscheius* species, the topology of the 28S tree shows that they could share a most recent common ancestor with representatives of the genus *Metarhabditis* (Tahseen et al., 2004). However, the 18S tree topology is inconsistent with that of the 28S relative to the more distantly positioned *Metarhabditis* in this deeper phylogenetic tree. This could be explained because small subunit rDNA sequences are better for elucidating higher relationships within organisms, phyla and classes, while large subunit sequences are useful for distinguishing among genera and species (Hillis and Dixon, 1991). Morphologically, both genera are superficially similar, differing in lip region (lips separated vs grouped in pairs), female rectum length (very long vs always short, as long as the anal body width), and bursa (three vs two well developed genital papillae at its posterior end. The long female rectum of *Oscheius* certainly is an apomorphic condition, and the paired lips and the presence of only two genital papillae in the bursa of *Metarhabditis* probably represent apomorphic states of their respective characters. Tabassum et al. (2016) transferred *Metarhabditis* species and its synonyms (Asif et al., 2013) to *Oscheius*, but the authors did not justify their action, which is not herein followed. The relationships with other genera of Rhabditidae remain more obscure as the branching of the tree is not definitely resolved.

Both morphological (Sudhaus and Hooper, 1994; Sudhaus, 2011) and molecular (among others Darby et al., 2011; Liu et al., 2012; Campos-Herrera et al., 2015; Torrini et al., 2015; Lima de Brida et al., 2017; Ye et al., 2018) evidences support the monophyly of *Oscheius*, with two well-defined monophyletic subgroups among its species. A reasonable translation of these ideas to classification results in the maintenance of *Oscheius* as valid genus, with *Dolichorhabditis* as its junior synonym, and the proposal of two subgenera: *Oscheius* for the *Insectivorus*-group of species and *Dolichorhabditis* for the *Dolichura*-group of species.

## Updated Taxonomy of *Oscheius*

In the following diagnoses of the genus and its two subgenera, a list of their species and a key to their identification are presented. The diagnoses are mainly based on Sudhaus’ (2011) ideas about the concept of the genus and the differences between the two species groups. In addition, the status of several species, in particular those described in recent years, is discussed.

### Oscheius Andrássy, 1976

=  *Rhabditis* (*Oscheius* Andrássy, 1976) Sudhaus, 1993

=  *Dolichorhabditis* Andrássy, 1983 (syn. by Sudhaus and Hooper, 1994)

=  *Heterorhabditidoides* Zhang et al., 2008 (syn. by Sudhaus, 2011)

#### Diagnosis

Rhabditidae. Small- to medium-sized nematodes, 0.50 to 3.25 mm long. Lateral field with three to five ridges (four to six incisures). Lip region continuous. Stoma tubular, bearing glottoid apparatus with small elongate teeth. Pharynx consisting of cylindrical corpus gradually enlarging posteriorly, with not swollen metacorpus, and basal bulb bearing duplex haustrulum. Secretory–excretory duct elongated, looped and strongly sclerotized. Female genital system didelphic–amphidelphic, with equatorial vulva. Female rectum conspicuously longer than anal body diameter, proximally dilated, forming a bladder-like expansion of the hind gut (often filled with faeces). Female tail conical to conical elongate. Testis reflexed ventrally. Bursa peloderan or leptoderan, anteriorly open, with wide velum bearing nine genital papillae arranged 1+1+1/3+3, GP5 and GP8 opening dorsally (bursa formula: v1,v2,v3/v4,ad,v5–v6,pd,v7,ph). Male tail conoid with or without a short acute terminal tip out of the bursa. Phasmid posterior to the last GP, tubular. Spicules free, dagger-shaped, head and slanted shoulder, the tip thickened.

### Subgenus Oscheius Andrássy, 1976

syn. *Oscheius sensu* Andrássy (1976, 1984, 2005).

(Fig. 6).

**Figure 6:**
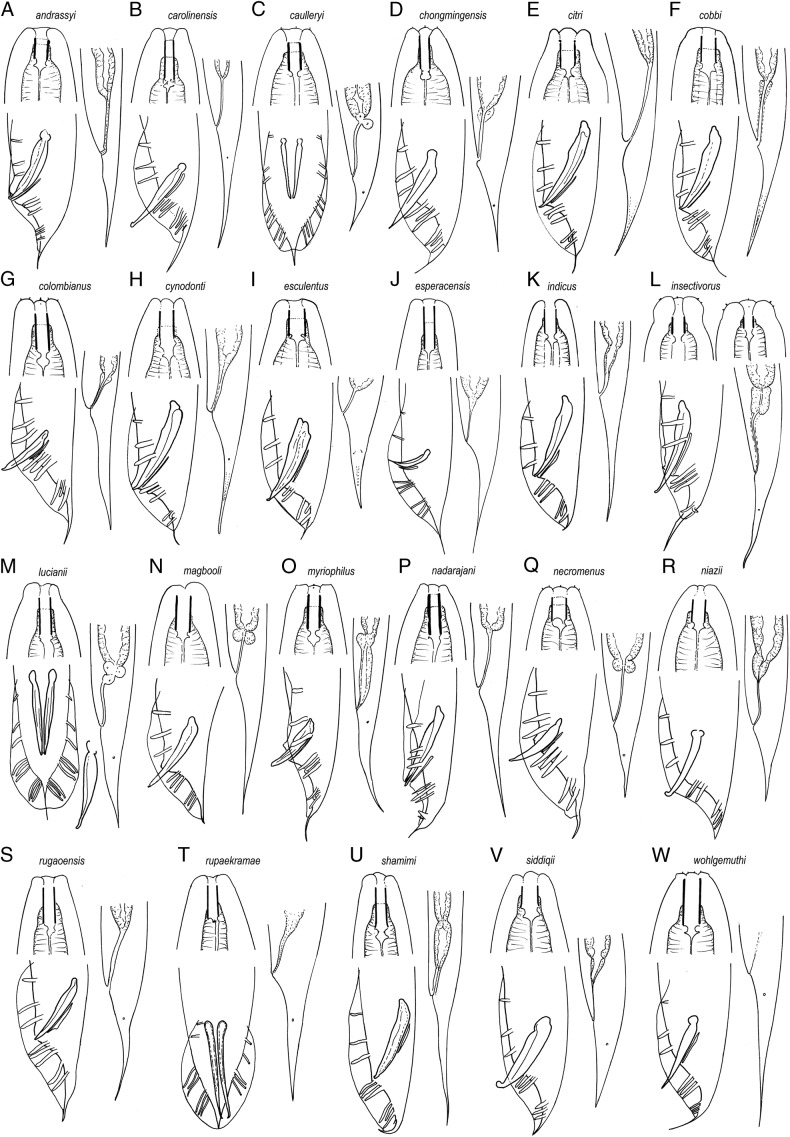
Lip region, male and female posterior ends of the species of the subgenus *Oscheius* (Andrássy, 1976) (not to scale). Based in the original illustrations (line drawings and LM pictures) except: isolated spicule in *O. lucianii* (cf. Chitwood, 1933).

#### Diagnosis

Stoma tubular with metastegostom bearing warts. Bursa leptoderan. Male tail conoid with tip out of the bursa, filiform, variable in length. Spicules distally hook-shaped, like a crochet needle.

#### Type species

Oscheius (Oscheius) insectivorus (Körner, 1954) Andrássy, 1976

 =  Rhabditis ***(Choriorhabditis)*** insectivora Körner, 1954

 =  Heterorhabditidoides (Oscheius) insectivora (Körner, 1954) Zhang et al., 2012

#### Other species

*O. (O.) andrassyi* Tabassum and Shahina, 2008

*O. (O.) carolinensis* Ye et al., 2010

 =  *Heterorhabditidoides (Oscheius) carolinensis* (Ye et al., 2010) Zhang et al., 2012

*O. (O.) caulleryi* (Maupas, 1919) Sudhaus and Hooper, 1994 (syn. by Andrássy, 2005)

 =  *Rhabditis caulleryi* Maupas, 1919

 =  *Rhabditis (Rhabditis) caulleryi* Maupas, 1919 (rank by Sudhaus and Schulte, 1989)

 =  *Rhabditis (Choriorhabditis) caulleryi* Maupas, 1919 (Osche, 1952)

 =  *Rhabditis (Oscheius) caulleryi* Maupas, 1919 (Sudhaus and Hooper, 1994)

*O. (O.) chongmingensis* (Zhang et al., 2008) Ye et al., 2010

 =  *Heterorhabditoides chongmingensis* Zhang et al., 2008

*O. (O.) citri* (Tabassum et al., 2016)*

*O. (O.) cobbi* (Tabassum et al., 2016)*

*O. (O.) colombianus* Stock et al., 2005 (rank by Andrássy, 2005)

 =  *Rhabditis (Oscheius) colombiana* Stock et al., 2005

 =  *Heterorhabditidoides (Oscheius) colombiana* (Stock et al., 2005) Zhang et al., 2012

*O. (O.) cynodonti* (Tabassum et al., 2016)*

*O. (O.) esculentus* (Tabassum et al., 2016)*

*O. (O.) esperancensis* (Stock, 1990) Sudhaus, 2011

 =  *Rhabditis esperancensis* Stock, 1990

*O. (O.) indicus* Kumar et al., 2019

*O. (O.) lucianii* (Maupas, 1919) Sudhaus and Hooper, 1994 (rank by Andrássy, 2005)

 =  *Rhabditis lucianii* Maupas, 1919

 =  *Rhabditis (Choriorhabditis) lucianii* Maupas, 1919 (Osche, 1952)

 =  *Rhabditis (Oscheius) lucianii* Maupas, 1919 (Sudhaus and Hooper, 1994)

*O. (O.) maqbooli* Tabassum and Shahina (2002)

*O. (O.) myriophilus* (Poinar, 1986) Sudhaus and Hooper, 1994 (rank by Sudhaus, 2011)

 =  *Rhabditis myriophila* Poinar, 1986

 =  *Rhabditis (Rhabditis) myriophila* Poinar, 1986 (rank by Sudhaus and Schulte, 1989)

 =  *Rhabditis (Oscheius) myriophila* Poinar, 1986 (Sudhaus and Hooper, 1994)

 =  *Heterorhabditidoides (Oscheius) myriophila* (Poinar, 1986) Zhang et al., 2012

*O. (O.) nadarajani* Ali et al., 2011

 =  *Oscheius punctatus* (Tabassum et al., 2016) (corrected name according to the ICZN) n. syn.^*^

 =  *Oscheius punctata* (Tabassum et al., 2016) (lapsus)

*O. (O.) necromenus* (Sudhaus and Schulte, 1989) Sudhaus and Hooper, 1994 (rank by Andrássy, 2005)

 =  *Rhabditis (Rhabditis) necromena* Sudhaus and Schulte, 1989

 =  *Rhabditis (Oscheius) necromena* Sudhaus and Schulte, 1989 (Sudhaus and Hooper, 1994)

*O. (O.) niazii* Tabassum and Shahina, 2010

*O. (O.) rugaoensis* (Zhang et al., 2012) (Darsouei et al., 2014)*

 =  *Heterorhabditidoides rugaoensis* Zhang et al., 2012

 =  *Dolichorhabditis dolichura apud* Tabassum and Shahina (2002) *nec* Schneider (1866) syn. n.*

*O. (O.) rupaekramae* (Khan et al., 2000) Sudhaus, 2011

 =  *Rhabditis rupaekramae* Khan et al., 2000

*O. (O.) shamimi* Tahseen and Nisa, 2006

 =  *Oscheius sacchari* (Tabassum et al., 2016) n. syn.*

*O. (O.) siddiqii* Tabassum and Shahina, 2010

*O. (O.) wohlgemuthi* (Völk, 1950) Tahseen and Nisa, 2006*

 =  *Rhabditis wohlgemuthi* Völk, 1950

 =  *Rhabditis (Choriorhabditis) wohlgemuthi* Völk, 1950 (Osche, 1952)

 =  *Rhabditis aspera apud* Örley (1886), *nec* Bütschli (1873)

### Subgenus Dolichorhabditis Andrássy, 1983 (n. rank)

syn. *Dolichorhabditis sensu* Andrássy (1983, 1984, 2005).

(Fig. 7).

**Figure 7:**
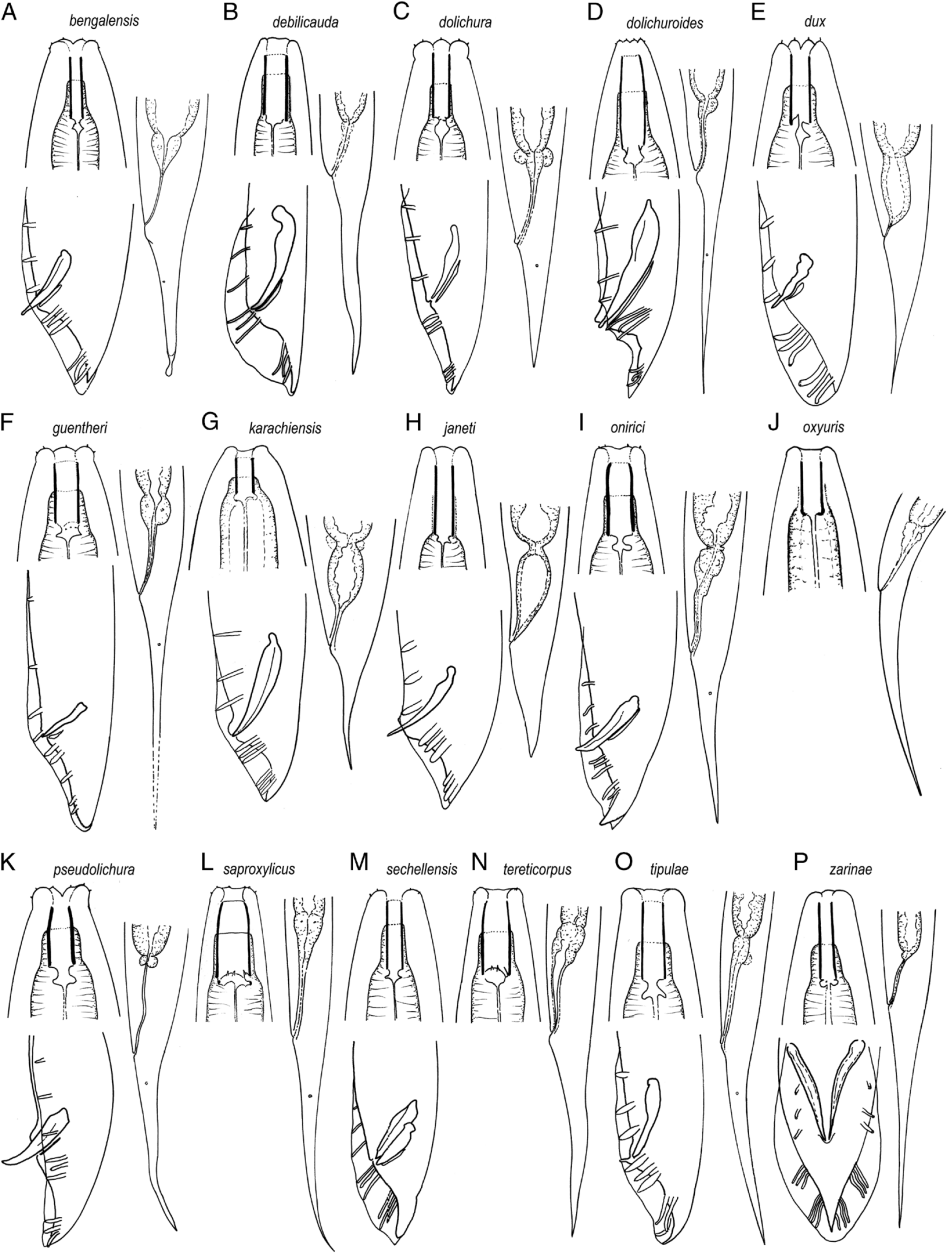
Lip region, male and female posterior ends of the species of the subgenus *Dolichorhabditis* (Andrássy, 1983) (not to scale). Based on the original illustrations (line drawings and LM pictures) except: *O. bengalensis* (cf. Sudhaus, 1974), *O. dolichura* (cf. Andrássy, 2005), lip region in *O. sechellensis* (cf. Sudhaus,1976), *O. tipulae* (cf. Abolafia and Peña-Santiago, 2010).

#### Diagnosis

Stoma tubular or barrel-shaped with metastegostom bearing setose teeth. Bursa peloderan. Male tail tip not reaching beyond bursa end. Spicule tips shaped like a probe head.

#### Type species

*Oscheius (Dolichorhabditis) dolichura* (Schneider, 1866) Sudhaus and Hooper, 1994 (n. rank)

 =  *Leptodera dolichura* Schneider, 1866

 =  *Rhabditis dolichura* (Schneider, 1866) Bütschli, 1873

 =  *Rhabditis (Caenorhabditis) dolichura* (Schneider, 1866) Bütschli, 1873 (Osche, 1952)

 =  *Caenorhabditis dolichura* (Schneider, 1866) Osche, 1952 (rank by Dougherty, 1955)

 =  *Rhabditis (Pellioditis) dolichura* (Schneider, 1866) Bütschli, 1873 (Sudhaus, 1976)

 =  *Rhabditis (Oscheius) dolichura* (Schneider, 1866) Bütschli, 1873 (Sudhaus and Hooper, 1994)

 =  *Dolichorhabditis dolichura* (Schneider, 1866) Andrássy, 1983

 =  *Rhabditis herfsi* Rahm, 1924

#### Other species

*O. (D.) bengalensis* (Timm, 1956) Sudhaus and Hooper, 1994 (n. rank)

 =  *Rhabditis (Choriorhabditis) marina bengalensis* Timm, 1956

 =  *Pellioditis marina bengalensis* (Timm, 1956) Timm, 1960

 =  *Rhabditis bengalensis* Timm, 1956

 =  *Rhabditis (Pellioditis) bengalensis* Timm, 1956 (Sudhaus, 1974)

 =  *Rhabditis (Oscheius) bengalensis* Timm, 1956 (Sudhaus and Hooper, 1994)

 =  *Oscheius bengalensis* Timm, 1956 (Sudhaus and Hooper, 1994) (rank by Sudhaus, 2011)

 =  *Dolichorhabditis bengalensis* (Timm, 1956) Andrássy, 2005

 =  *Rhabditis bengalensis mexicana* Hopper, 1963

*O. (D). karachiensis* (Mehmood and Khanum, 2018) (n. comb., n. rank)

 =  *Oscheius karachiensis* Mehmood and Khanum, 2018

*O. (D.) debilicauda* (Fuchs, 1937) n. comb. (n. rank)^*^

 =  *Rhabditis debilicauda* Fuchs, 1937

 =  *Rhabditis (Caenorhabditis) debilicauda* Fuchs, 1937 (Osche, 1952)

 =  *Caenorhabditis debilicauda* (Fuchs, 1937) Osche, 1952 (rank by Dougherty, 1955)

 =  *Dolichorhabditis debilicauda* (Fuchs, 1937) Andrássy, 1983

*O. (D.) dolichuroides* (Anderson and Sudhaus, 1985) Sudhaus and Hooper, 1994 (n. rank)

 =  *Rhabditis (Pellioditis) dolichuroides* Anderson and Sudhaus, 1985

 =  *Rhabditis (Oscheius) dolichuroides* Anderson and Sudhaus, 1985 (Sudhaus and Hooper, 1994)

*=  Oscheius dolichuroides* Anderson and Sudhaus, 1985 (Sudhaus and Hooper, 1994) (rank by Sudhaus, 2011)

 =  *Dolichorhabditis dolichuroides* (Anderson and Sudhaus, 1985) Andrássy, 2005

*O. (D.) dux* (Gorgadze, 2010) Sudhaus, 2011* (n. rank)

 =  *Dolichorhabditis dux* Gorgadze, 2010

 =  *Oscheius dux* (Gorgadze, 2010) Sudhaus, 2011

*O. (D.) guentheri* (Sudhaus and Hooper, 1994) Andrássy, 2005 (n. rank)

 =  *Dolichorhabditis guentheri* (Sudhaus and Hooper, 1994) Andrássy, 2005

*=  Oscheius guentheri* (Sudhaus and Hooper, 1994) Andrássy, 2005

*O. (D.) janeti* (Lacaze-Duthiers in Janet, 1893) Sudhaus, 2011 (n. comb.)

 =  *Pelodera janeti* De Lacaze-Duthiers in Janet, 1893 (*nomen nudum*; species described by Janet, 1894)

 =  *Rhabditis janeti* (De Lacaze-Duthiers in Janet, 1893) de Man, 1894

 =  *Rhabditis (Protorhabditis) janeti* (De Lacaze-Duthiers in Janet, 1893) de Man, 1894 (Osche, 1952)

*=  Oscheius janeti* (Lacaze-Duthiers in Janet, 1893) Sudhaus, 2011

*O. (D.) latus* (Cobb, 1906) Sudhaus, 2011 (n. comb.)

 =  *Rhabditis latus* Cobb, 1906

*=  Oscheius latus* (Cobb, 1906) Sudhaus, 2011

*O. (D.) onirici* Torrini et al., 2015* (n. comb.)

 =  *Oscheius onirici* Torrini et al., 2015

 =  *Oscheius tipulae* apud Abolafia and Lechuga-Puñal (2014), nec Lam and Webster (1971)

*O. (D.) pseudodolichura* (Körner in Osche, 1952) Sudhaus and Hooper, 1994 (n. rank)

 =  *Rhabditis (Caenorhabditis) pseudodolichura* Körner in Osche, 1952 (described by Körner, 1954)

 =  *Caenorhabditis pseudodolichura* Körner in Osche, 1952 (rank by Dougherty, 1955)

 =  *Pellioditis pseudololichura* (Körner in Osche, 1952) Andrássy, 1983

 =  *Rhabditis (Oscheius) pseudodolichura* Körner in Osche, 1952 (Sudhaus and Hooper, 1994)

 =  *Oscheius pseudodolichura* Körner in Osche, 1952 (Sudhaus and Hooper, 1994) (rank by Sudhaus, 2011)

 =  *Rhabditis (Choriorhabditis) pseudodolichura* Körner in Osche, 1952 (Mengert, 1953)

 =  *Dolichorhabditis pseudodolichura* (Körner in Osche, 1952) Andrássy, 2005

*O. (D.) saproxylicus* sp. n.

*O. (D.) sechellensis* (Potts, 1910) Sudhaus and Hooper, 1994 (n. rank)

 =  *Rhabditis sechellensis* Potts, 1910

 =  *Rhabditis (Choriorhabditis) sechellensis* Potts, 1910 (Osche, 1952)

 =  *Rhabditis (Pellioditis) sechellensis* Potts, 1910 (Sudhaus, 1976)

 =  *Rhabditis (Oscheius) sechellensis* Potts, 1910 (Sudhaus and Hooper, 1994)

*=  Oscheius sechellensis* (Potts, 1910) Sudhaus and Hooper, 1994 (rank by Sudhaus, 2011)

 =  *Dolichorhabditis sechellensis* (Potts, 1910) Andrássy, 2005

*=  Oscheius sechellensis* (Potts, 1910) Sudhaus and Hooper, 1994 (rank by Sudhaus, 2011)

*O. (D.) tereticorpus* (Kito and Ohyama, 2008) Sudhaus, 2011 (n. rank)

 =  *Dolichorhabditis tereticorpus* Kito and Ohyama, 2008

*=  Oscheius tereticorpus* (Kito and Ohyama, 2008) Sudhaus, 2011

*O. (D.) tipulae* Lam and Webster, 1971* (n. rank)

 =  *Rhabditis (Rhabditella) tipulae* Lam and Webster, 1971

 =  *Rhabditis (Oscheius) tipulae* Lam and Webster, 1971 (Sudhaus, 1993)

 =  *Oscheius tipulae* Lam and Webster, 1971 (rank by Sudhaus, 2011)

 =  *Dolichorhabditis tipulae* (Lam and Webster, 1971) Andrássy, 2005

*O. (D.) zarinae* (Khan et al., 2000) Sudhaus, 2011* (n. rank)

 =  *Rhabditis zarinae* Khan et al., 2000

 =  *Oscheius zarinae* (Khan et al., 2000) Sudhaus, 2011

#### Species inquirendae

*O. (D.). oxyuris* (Claus, 1862) n. comb. (n. rank)*

 =  *Anguillula oxyuris* Claus, 1862, nec *Rhabditis oxyuris* apud Bütschli (1873)

#### citri

Very similar to *O. andrassyi*, from which it only differs in its longer spicules (57–70 vs 45–51 µm).

#### cobbi

Nearly identical to *O. siddiqii*, but distinguishable from this by its shorter male tail (20–32 vs 38–45 µm length).

#### cynodonti

Much resembling *O. rupraekramae*, it can be separated from this in its smaller general size (female body 1.25–1.66 vs 1.80–2.40 mm long). Female tail in the former is mentioned to be comparatively longer (*c*’ = 4.0–6.7 (3.1 in LM picture and 3.8 in drawing) vs 2.6–3.7), but *c*’ is ca. 3.1 in LM picture and 3.8 in line drawing, thus raising a doubt about the consistency of this difference.

#### debilicauda

The identity of this species has raised some controversy as Andrássy (2005) considered it as a valid species whereas Sudhaus (2011) regarded it as junior synonym of *O. dolichura*. Both taxa are distinguishable by their female rectum length (slightly longer than vs ca. 1.5–2.0 times the anal body diam.), female tail length (*c*’ = 3–4 vs *c*’ = 4.7), morphology of spicules (manubrium rounded, swollen and strongly bent ventrad vs rounded but not swollen and slightly bent ventrad), and genital papillae arrangement (GP4-6 at level of vs posterior to cloacal). Thus, they are herein kept as valid and separate species.

#### dolichura apud

Tabassum and Shahina (2002), *nec* Schneider (1866): This material is not certainly conspecific with other populations of *O. (D.) dolichura* due to relevant differences in female rectum (3 vs 1.5 anal body widths long), and much longer spicules (46–74 vs 23–35 µm) exceeding (vs not reaching) the GP1. It better fits the *O. rugaoensis* diagnosis.

#### dux

Very similar morphologically to *O. janeti*, both species only differ in some aspects of their reproductive biology, as the former presents hermaphroditic females and very rare males whereas the latter have equally abundant females and males.

#### esculentus

Similar to *O. magbooli*, both species differ in their general size (female body 1.25–1.80 vs 0.94–1.34 mm long), more posterior location of excretory pore (at level of basal bulb vs at posterior part of isthmus), female tail slightly shorter (90–120 µm, *c* = 13.0–15.3, *c*’ =  2.9–4.2 vs 112–148 µm, *c* = 6.7–11.0, *c*’ = 4.6–5.9), and slightly shorter gubernaculum (20–24 vs 26–30 µm).

#### onirici

Morphologically, this species and *O. tipulae* are near indistinguishable, but molecular analyses show relevant differences. Thus, they might represent a case of cryptic species within the subgenus *Dolichorhabditis*.

#### oxyuris

The identity of this species maintains some doubts being regarded by Andrássy (1983) as junior synonym of *O. dolichura*. However, some morphological characters as lip region not offset and female tail morphology (very thin and elongate, *c*’ = 7.6) do not agree with the type description of *O. dolichura*. Probably senior synonym of *O. onirici* or *O. tipulae*.

#### punctatus

Nearly identical to *O. nadarajani*, it can be distinguished from this in stoma length (10–18 vs 18–19 µm) and excretory pore position (basal bulb vs isthmus). Nevertheless, these differences are so minor that they do not justify its separation. Thus, *O. punctatus* is herein regarded as a junior synonym of *O. nadarajani.*

#### rugaoensis

Originally described as belonging to *Heterorhaditidoides* by Zhang et al. (2012), Darsouei et al. (2014) mentioned this species as *Oscheius rugaoensis*, but these authors did not justify the nomenclatorial change.

#### sacchari

Very similar to *O. shamimi,* both species are distinguishable by minor and questionable differences: slightly larger females (1.36–2.02 vs 0.76–1.52 mm), slightly posterior position of the excretory pore (at basal bulb level vs at isthmus level), and slightly shorter spicules (47–55 vs 53–67 µm) and gubernaculum (18–22 vs 22–28 µm), insufficient differences to maintain them as separate species.

#### tipulae

Morphologically, this species and *O. onirici* are nearly indistinguishable, but molecular analyses show relevant differences. Thus, they represent a case of cryptic species within the subgenus *Dolichorhabditis*.

#### wohlgemuthi

Tahseen and Nisa (2006) mentioned this species as *Oscheius wohgelmuthi*, but they did not justify the nomenclatorial change which was later officially promoted by Sudhaus (2011). On the other hand, the morphological pattern of this species, with swollen metacorpus (spheroid), moderate length rectum (1.2–1.6 times ABW) and male with bursa bearing nine genital papillae with GP1 and GP2 more widely spaced than GP2 and GP3 (1+2/3+3), agrees better with the members of the *Brassicae*-group (Sudhaus, 2011) of the genus *Rhabditis* (Dujardin, 1845).

#### zarinae

Very similar to *O. debilicauda*, both species being distinguishable in a few minor (but apparently relevant) differences in their general size (body 0.75–1.56 vs 0.53–0.75 mm long), female tail shape (posterior half very thin, almost filiform vs thicker, not filiform), and spicule length (32–48 vs 29 µm).

#### Key to species identification

1a – Body length very long, 6 mm *latus*

1b – Body length shorter, less than 3.5 mm 2

2a – Stoma barrel-shaped or tubular; bursa peloderan (unknown in O*. tereticorpus* and *O. saproxylicus* sp. n.) 3

2b – Stoma tubular; bursa leptoderan or pseudopeloderan 16

3a – Each spicule visibly with different size *sechellensis*

3b – Both spicules with similar size 4

4a – Female rectum scarcely longer than the anal diameter 5

4b – Female rectum ca. 2 to 3 times longer than the anal diameter 6

5a – Body length less than 750 µm; spicules 29 µm long *debilicauda*

5b – Body length more than 750 µm; spicules 32 to 48 µm long *zarinae*

6a – Female tail short conoid (*c*’ < 4) 7

6b – Female tail longer, elongate (*c*’ > 4, rarely 3) 9

7a – Lip region slightly offset by depression; female rectum longer, 3 times anal body diam. *dolichura*

7b – Lip region not offset; female rectum shorter, 2 times anal body diam. 8

8a – Males as frequent as females; spicules longer, 35 µm, exceeding the GP1 *janeti*

8b – Males very rare; spicules shorter, 22 to 30 µm, not reaching the GP1 *dux*

9a – Lip region visibly narrower than adjacent part of body; spicules longer, more than 40 µm 10

9b – Lip region equal or wider than adjacent part of body; spicules shorter, less than 30 µm (unknown in *O. tereticorpus* and *O. saproxylicus* sp. n.) 11

10a – Male body more than 1mm long; spicules longer, 41 to 48 µm *dolichuroides*

10b – Male body less than 1 mm long; spicules shorter, 50 to 52 µm *karachiensis*

11a – Gymnostom anteriorly narrower, with convex walls 12

11b – Gymnostom with parallel walls 14

12a – Lip region offset by constriction; metacorpus slightly swollen *pseudodolichura*

12b – Lip region not offset or slightly offset by depression; metacorpus not swollen 13

13a – Lip region slightly offset by depression; gymnostom as long as promesostegostom; pharynx with metacorpus with sclerotized walls, valves-like; spermatheca differentiated in a sac *tereticorpus*

13b – Lip region not offset; gymnostom slightly shorter than promesostegostom; pharynx with metacorpus without sclerotized walls; spermatheca not differentiated in a sac *saproxylicus* sp. n.

14a – Lip region slightly offset; female rectum ca. two times longer than ABW; GP1 very anterior, outside of the range of the spicules *guentheri*

14b – Lip region not offset; female rectum ca. three times longer than ABW; GP1 at spicules level 15

15a – Body length slightly larger (584–801 µm long); neck slightly shorter relative to the body length (*b* = 4.4–6.0); female tail slightly shorter (63–81 µm, *c* = 8.6–11.8, *c*’ = 3.5–5.0) *onirici*

15b – Body length slightly smaller (505–691 µm long); neck slightly longer relative to the body length (*b* = 3.9 to 4.9); female tail slightly longer (70–95 µm, *c* = 6.2–8.5, *c*’ = 4.2 to 6.4) *tipulae*

16a – Female rectum ca. as long or slightly longer than anal body width 17

16b – Female rectum longer than body width 22

17a – GP1 very reduced 18

17b – All GPs with similar size 20

18a – Spicules distally straight *wohlgemuthi*

18b – Spicules distally slightly hook-like 19

19a – Female body less than 1.7 mm long *cynodonti*

19b – Female body more than 1.8 mm long *rupraekramae*

20a – Female stoma 21 to 28 µm long *colombianus*

20b – Female stoma 12 to 20 µm long 21

21a – Female body 0.9 to 1.3 mm long; female tail more slender (*c*’ = 4.6-5.9) *magbooli*

21b – Female body 1.3 to 1.8 mm long; female tail shorter (*c*’ = 2.9-4.2) *esculentus*

22a – Stomatal tube shorter, ca. 1.0 to 2.0 times longer than wide 23

22b – Stomatal tube longer, ca. 2.5 to 3.0 times longer than wide 30

23a – Cheilostom as long as stomatal tube length *insectivorus*

23b – Cheilostom one third of the stomatal tube length 24

24a – GP1 very separated from GP2, and GP2-3 very close *caulleryi*

24b – GP1-2 distance similar or slightly more than GP2-3 distance 25

25a – Spicules with ventral bent tip 26

25b – Spicules with thin tip, crochet needle-like 28

26a – Spicules 43 to 52 µm, with thin tip; GP1-2 distance similar to GP2-3 distance *niazii*

26b – Spicules 50 to 62 µm, with thick tip; GP1-2 distance slightly more than GP2-3 distance 27

27a – Male tail shorter (20–32 µm long) *cobbi*

27b – Male tail longer (38–45 µm long) *siddiqii*

28a – Female rectum ca. 1.5 times longer than ABW; spicules shorter, 34 to 44 µm long *necromenus*

28b – Female rectum ca. 2.0–2.5 times longer than ABW; spicules longer, 45 to 70µm long 29

29b – Spicules shorter (45–51 µm long) *andrassyi*

29a – Spicules longer (57–70 µm long) *citri*

30a – Spicules ventrad curved, as long as anal body width *esperacensis*

30b – Spicules almost straight, longer than anal body width 31

31a – Spicules ca. 1.5 times longer than the gubernaculum *myriophilus*

31b – Spicules ca. 2–3 times longer than the gubernaculum 32

32a – Spicules with manubrium longer than wide 33

32b – Spicules with manubrium as long as wide 35

33a – Lip region higher, twice wider than high *lucianii*

33b – Lip region lower, three times wider than high 34

34a – Stoma shorter, 9 to 10 µm; female tail shorter, *c*’ = 3.1–4.8, rarely longer) *chongmingensis*

34b – Stoma longer, 13 to 18 µm; female tail longer, *c*’ = 5.0–6.6) *indicus*

35a – Female rectum ca. 3.0 to 4.5 times longer than anal body width *shamimi*

35b – Female rectum ca. 1.5 times longer than anal body width 36

36a – Female tail shorter, *c*’ = 2.2–3.1 *rugaoensis*

36b – Female tail longer, *c*’ = 3.3–6.7 *nadarajani*

### Other nominal species of Oscheius not included in the key

#### microvilli

Zhou et al. (2017) recently described this species, mainly based on molecular analyses, but a reasonable doubt persists about its true identity. Three morphological features of its diagnosis better fit the pattern observed in *Caenorhabditis* Osche, 1952: swollen, somewhat spheroid metacorpus (vs not swollen or slightly fusiform in *Oscheius*), genital papillae arranged in the form 2/1+3+3 (vs 1+1+1/3+3 or 1+2/3+3), and anteriorly closed bursa with irregular margin (vs open bursa with smooth margin). Original material might also consist of more than one species as line illustrations of female, especially its long rectum and conical tail probably correspond to some species of *Oscheius*, whereas the illustrations of male (and probably the female examined by SEM too) resemble those of some *Caenorhabditis* representatives, in particular *C. sinica* (Huang et al., 2014). Molecular data show a close relationship with *Oscheius* species. Until new information was available, *O. microvilli* is regarded as *species inquirenda.*

#### pheropsophi

Originally described as *Rhabditis (Oscheius) pheropsophi* by Smart and Nguyen (1994), this species is characterized by having fused spicules, a totally unusual feature in *Oscheius* species, which only occurs in representatives of the superfamily Mesorhabditoidea (Andrássy, 1976) *sensu* (De Ley and Blaxter, 2002, 2004). It resembles the species belonging to the *Teres*-group of the genus *Pelodera* Schneider, 1866 (see Andrássy, 2005; Shokoohi and Abolafia, 2011) in having swollen pharyngeal corpus and ten (1+2/3+4) genital papillae too. Thus, it is herein provisionally regarded as *species incertae sedis*.

## Footnotes

1. The specific epithet refers to the habitat where the species was found, decaying wood.
